# Maternal separation is associated with strain-specific responses to stress and epigenetic alterations to *Nr3c1, Avp*, and *Nr4a1* in mouse

**DOI:** 10.1002/brb3.69

**Published:** 2012-06-29

**Authors:** R L Kember, E L Dempster, T H A Lee, L C Schalkwyk, J Mill, C Fernandes

**Affiliations:** 1 Social, Genetic and Developmental Psychiatry Centre, Institute of Psychiatry, King's College LondonDe Crespigny Park, London, UK; 2 Department of Neuroscience, Institute of Psychiatry, King's College LondonDe Crespigny Park, London, UK

**Keywords:** Behavior, epigenetics, genetics, maternal separation, mouse, stress

## Abstract

Stressful events early in life have been widely linked to behavioral phenotypes and have been implicated in the development of psychiatric disorders. Using a maternal separation paradigm, we investigated phenotypic and epigenetic changes following early life stress in two inbred strains of mice, C57BL/6J and DBA/2J. We found an increase in the corticosterone response to stress in male, C57BL/6J mice that had undergone maternal separation compared to controls. In addition, early life stress induced a number of mild but significant behavioral changes, many of which were sex and strain dependent. Following maternal separation anxiety was decreased in males but increased in DBA/2J females, DBA/2J males displayed reduced exploration of a novel object, and baseline activity was altered in males of both strains. Finally, we examined DNA methylation levels in the hippocampus across promoter regions of *Nr3c1, Avp*, and *Nr4a1*, and found altered levels at several CpG sites in maternally separated male mice compared to controls. This study contributes to a growing body of recent literature suggesting that epigenetic changes may mediate the impact of early life stress on behavior. In particular, we establish that the phenotypic and epigenetic responses to an adverse environment differ as a function of genetic background.

## Introduction

Stressful events early in life have been widely linked to behavioral phenotypes and have been implicated in the development of psychiatric disorders (Heim and Nemeroff 2001; Gilman et al. 2003). For instance, early life adversity appears to play a major role in the etiology of depression (Gillespie et al. 2005), and hormones involved in the stress response, including corticotrophin-releasing hormone (CRH) and cortisol, have been shown to be elevated in depressed individuals (Gibbons and Mchugh 1962; Merali et al. 2004). Clearly, not everyone experiencing stress early in life becomes depressed and it has been suggested that genetic factors influence susceptibility or resilience to the adverse effects of early life stress (Caspi et al. 2003; Heim et al. 2008). Mounting evidence suggests that these gene–environment interactions (G × E) may be mediated by epigenetic mechanisms operating at the interface between the genome and the environment (Dolinoy et al. 2007). Changes in DNA methylation following early life stress have been associated with long-term changes in gene expression and behavior (Champagne and Curley 2009) and may contribute to psychiatric disorders (Rutten and Mill 2009) and physiological disturbances (Gluckman et al. 2007) later in life.

Recent research using rodent models provides direct evidence for the role of early life stress on the epigenome. Weaver et al. (2004) observed that poor maternal care in rats alters DNA methylation at a specific sequence motif upstream of the glucocorticoid receptor gene (*Nr3c1*) in the hippocampus of the offspring, directly affecting transcription and subsequent stress responses in adulthood. However, unchanged DNA methylation in the same region of *Nr3c1* in the hippocampus following a model of maternal separation (MS) in rat has also been reported (Daniels et al. 2009), highlighting the need to confirm the link between early life stress and epigenetic alterations at this locus. Early life stress has been shown to bring about epigenetic changes at the arginine vasopressin gene (*Avp*), with a regulatory region in the gene being hypomethylated following MS (Murgatroyd et al. 2009). Similar changes following an environmental stressor have been observed in several other genes including *Bdnf* (Fuchikami et al. 2009; Roth et al. 2009), *Crh* (Elliott et al. 2010), *Dlgap2* (Chertkow-Deutsher et al. 2010), *Mecp2, Cnr1,* and *Crhr2* (Franklin et al. 2010), suggesting that such changes may occur in multiple neurobiological pathways in response to stress.

In this study, our aim was to explore physiological, behavioral, and epigenetic changes in response to early life stress in the mouse, and determine whether these differed as a function of genetic background. We used MS, a validated model of early postnatal life stress in rodents, that is known to induce long lasting effects on emotional behavior and stress-reactivity (Boccia and Pedersen 2001; Holmes et al. 2005), changes to the hypothalamic–pituitary–adrenocortical (HPA) axis (Schmidt et al. 2004), and result in a significant loss of neurons in the hippocampus of adult mice (Fabricius et al. 2008). MS models vary in the literature both in the frequency and in the length of separation, which has led to a disparity in phenotypic changes seen. We chose to use the single 24 h separation model to avoid the phenotypic variability found in repeated separation models, as the length of the separation period seems to mediate whether a positive or negative behavioral change is seen (Holmes et al. 2005), possibly due to the increase of maternal care after the separation (Millstein and Holmes 2007).

Corticosterone levels in response to a stress challenge and a range of behavioral phenotypes were measured in adult mice following MS. DNA methylation levels in the promoter regions of three candidate genes in two strains of inbred mouse (C57BL/6J and DBA/2J) following MS were determined; based on previous studies we chose *Nr3c1* and *Avp* as likely targets of early life stress, and *Nr4a1*, encoding a brain-expressed nuclear hormone receptor, was selected given its involvement in disorders such as schizophrenia and depression.

## Methods

### Animals

C57BL/6J and DBA/2J mice were bred in the Biological Services Unit at the Institute of Psychiatry, Kings College London using original stocks [respective stock numbers: 000664, 000671] purchased from The Jackson Laboratory (Bar Harbor, ME). DBA/2J and C57BL/6J strains were selected as these represent members of a priority list based on the most well-characterized, commonly used strains for gene manipulation and crosses (Mouse Phenome Project, http://aretha.jax.org/pub-cgi/phenome/mpdcgi?rtn=docs/home).

Mice were housed in standard cages measuring 30.5 × 13 × 11 cm, kept at an ambient temperature (21 ± 2°C) and light (light/dark cycle with white lights on from 08:00 to 20:00), with food (Rat and Mouse No. 3 diet, Special Diet Services, Essex, UK) and tap water available ad libitum. Sawdust (Litaspen premium) and nesting materials (Sizzlenest; Datsand, Manchester, UK) in each cage were changed once every 2 weeks, but never on the day before or the day of testing to minimize the disruptive effect of cage cleaning on behavior. All housing and experimental procedures were performed in compliance with the UK Home Office Animals Scientific Procedures Act 1986.

### Early life stress

To model early life stress, a MS protocol was used. Males were paired with female breeders for 2 weeks and then removed. Litters of each strain were randomly allocated to control or maternal separation (MS) groups. For the litters in the MS group, the mother was removed from the litter on postnatal day 9 for 24 h and returned to the housing room, leaving the pups undisturbed. The cages containing the litters were placed on a heating pad and kept in a procedure room to maximize separation from their mother. After 24 h, the dam was returned to the litter and the cage returned to the housing room. (Control group litters were not disturbed and remained in the housing room with their mothers until they were weaned.) Mice were weaned aged 5 weeks and two pups within each litter were randomly assigned to one of three groups; test-naïve adolescent group (culled at 5 weeks), test-naïve adults (culled at 14–15 weeks), and test adults (tested at 11–12 weeks, culled at 14–15 weeks). The groups of adult mice were transferred at approximately 9 weeks of age to a separate housing and test facility and pair housed with a same sex sibling. All mice were allowed to habituate for 2 weeks before being either culled or undergoing a battery of behavioral tests and then culled.

### Behavioral tests

A battery of behavioral tests were conducted in the following order: home cage activity, open field, novel object exploration, holeboard, and forced swim test. Specific details of each test are given below. Offspring were aged 11 to 12 weeks at the start of testing (total *n* = 84). Group sizes were as follows (*n* = 4–7 litters/group): C57BL/6J control (male *n* = 14, female *n* = 8), C57BL/6J separated (male *n* = 10, female *n* = 10), DBA/2J control (male *n* = 10, female *n* = 12), and DBA/2J separated (male *n* = 10, female *n* = 10). Behavioral tests were performed during the light cycle between 09:00 and 18:00 h; except for the home cage in which mice were tested between 01:00 and 02:00 for the dark phase hour. Each apparatus was wiped clean with 1% Trigene® between subjects to avoid olfactory cueing behaviors. Behaviors for all tests were recorded on videotapes for further detailed analysis. Mice were returned to their home cage at the end of each test.

#### Home cage

Animals were tested in groups of four using adapted breeding cages (40 × 25 × 12 cm); this test was designed to observe spontaneous behaviors in a home cage-like environment (Lad et al. 2010). Methods are identical to those reported previously (Lad et al. 2010), except for the timing of the test. Mice were transferred to these home cages during the middle of their light cycle (between 12:30 and 13:00). Recording took place at three time points during the 24 h test (13:00–14:00; 01:00–02:00; 11:30–12:30). The first hour (13:00–14:00) following the transfer of the mice measured their behavior in response to the novel environment. The second hour (01:00–02:00) assessed their behavior during the dark phase (the active phase for mice which are nocturnal mammals), following 12 h of habituation. The last hour (11:30–12:30) measured the behavior of the mice following an extended period (>22 h) of habituation during a typically low activity phase (light phase). Four red multi-LED cluster lamps (LED cluster red light No. 310-6757; RS Components Northants, UK) of approximate wavelength 705 nm were placed in the test room to provide sufficient lighting for the image capture, but give the appearance of darkness to the mice given the wavelength of the lamps.

#### Open field

The open field was performed as described previously (Lad et al. 2010), except for light level which was ∼30 lux.

#### Novel object exploration

Novel object exploration was performed 48 h after the open field test using the open field arena (see Lad et al. 2010 for details). During the novel object exploration task, each mouse was exposed to two identical novel objects for 5 min. The Ethovision program was utilized for both automated tracking and manual scoring. Manual scoring allowed accurate measures of exploration (frequency and duration) of each object to be made.

#### Holeboard

The holeboard (File 2001), used to measure activity and exploration in a novel arena, was run in a Tru Scan Photo Beam Activity System (Coulbourn Instruments, Allentown, New Jersey), which consisted of an automated arena (25.4 × 25.4 × 40.6 cm) with sensor rings to track movement in the arena (light level 300 lux). The beams were spaced 1.52 cm apart providing a 0.76 cm spatial resolution. A metal floor was used, containing 16 holes (2.2 cm in diameter), evenly distributed over the floor (4 × 4 configuration). The floor also contained sensors to detect nose pokes. Mice were placed individually in a corner of the holeboard and allowed to freely explore for 5 min. The distance traveled, number of holes visited, and time spent in the center (17.8 × 17.8 cm) were recorded using the Tru Scan Software Version 2.0 (Coulbourn Instruments).

#### Forced swim

The forced swim test (Porsolt et al. 1978) was carried out in a clear Perspex tube (49 cm high × 15 cm diameter) filled with water at room temperature (depth 40 cm). Twenty-four hours prior to the trial, a blood sample was taken to provide a baseline measure of corticosterone, see below. The animal was placed in the water and left to swim for 6 min. Behavior in the last 4 min of the test was manually scored using the Ethovision program, and defined as either swimming (movement) or immobility (the absence of movement except that necessary to keep afloat). Thirty minutes after the forced swim test, a second blood sample was taken to measure the corticosterone level post-swim stress.

### Corticosterone assay

Approximately 50 μL of whole blood was collected exactly 24 h prior to forced swim testing (baseline or pre-stress measure) and again 30 min after the first forced swim trial (post-stress measure) from the tail vein by nicking the tail without restraint of the mouse. Blood collection was completed within 120 sec after removing each mouse from its cage. Blood was collected into potassium-EDTA microvette CB 300 tubes (Sarstedt, Nümbrecht, Germany). Plasma corticosterone levels were determined in duplicate from 20 μL of plasma using commercially available enzyme immunoassay kits (Assay Designs, Ann Arbor, Maine); sensitivity 30 pg/mL.

### Epigenetic analysis

#### Sample preparation

As the male mice produced the most robust behavioral changes following early life stress, we chose to focus on them for the epigenetic part of the study. Mice were killed by cervical dislocation and the hippocampus, a key area of the brain involved in behaviors, such as anxiety, aggression and learning and memory (Fernandes et al. 2004), was dissected from 14- to 15-week-old male C57BL/6J control (*n* = 10), C57BL/6J separated (*n* = 8), DBA/2J control (*n* = 12) and DBA/2J separated (*n* = 6) behaviorally naïve mice (total *n* = 36). DNA was extracted from the tissue using the Qiagen AllPrep DNA/RNA kit (Crawley, UK), using the manufacturer's standard protocol. All DNA samples were quantified and quality tested.

#### DNA methylation analysis

Genomic DNA (400 ng) was treated with sodium bisulfite using the EZ-96 DNA Methylation Kit (Zymo Research, Irvine, California) following the manufacturer's standard protocol. DNA methylation was quantitatively assessed using the Sequenom EpiTYPER system (Sequenom Inc., San Diego, California) as described previously (Ehrich et al. 2005). Bisulfite-PCR amplicons were designed to span CpG sites in promoter regions of *Nr3c1*, *Avp,* and *Nr4a1*. Primer sets, locations, and PCR conditions for each region are presented in Table 1. Positive controls, including both artificially methylated and artificially unmethylated samples were included in all experimental procedures to ensure unambiguous PCR amplification of bisulfite-treated samples. Each sample was processed in duplicate to reduce technical variance, with the correlation between technical duplicates being 0.95 across all assays. The data presented are the average of duplicate runs. Data generated from the EpiTYPER software were filtered using stringent quality control parameters, and CpG units with low call rates and/or individuals with a high number of missing CpG units were removed.

**Table 1:** Primer sequences, location, and PCR conditions

Amplicon	Primer sequence	Location	PCR conditions
Avp 1	F: 5′-GGAGTAGAAGGTATTTTTGGTTTGAA-3′	Chr2: 130405331–130405668	47 cycles, annealing temperature of 57°C
	R: 5′-CCAAACACACACATAATACCCAAAT-3′		
Avp 2	F: 5′-AGGTAGGTTATTGGTGGATAAAAGG-3′	Chr2: 130405615–130406036	47 cycles, annealing temperature of 60°C
	R: 5′-TTCCATCTCCATAATACTAAAAACCA-3′		
Nr4a1	F: 5′-GTTATTTTTAGTTTATTGATGAGGTTG-3′	Chr15: 101084504–101085001	47 cycles, annealing temperature of 56°C
	R: 5′-AAAAATTCATCCATACAAACCACC-3′		
Nr3c1	F: 5′-GGTGGGTTTTGTTTTGTAATTTTTT-3′	Chr18: 39648790–39651207	49 cycles, annealing temperature of 61°C
	R: 5′-AATTTCTTTAATTTCTCTTCTCCCTAA-3′		

### Statistical analysis

Data analysis was performed using R (http://www.R-project.org). For the behavioral data, a qualitative comparison between sexes was made, and sexes were then analyzed separately as the epigenetic work was conducted exclusively in males. Independent factors were: Strain (G; C57BL/6J and DBA/2J) and Environment (E; MS or control). The data were analyzed with a factorial analysis of variance (ANOVA) to determine the significance of the main factors (strain and environment as fixed factors) and any interaction between the main factors. For the epigenetic data, differences in DNA methylation were analyzed by two-tailed unpaired *t*-test for each CpG unit between groups within a given strain. In all cases, the nominal level of significance was *P* < 0.05.

## Results

### Behavioral changes in response to MS

**Figure 1:**
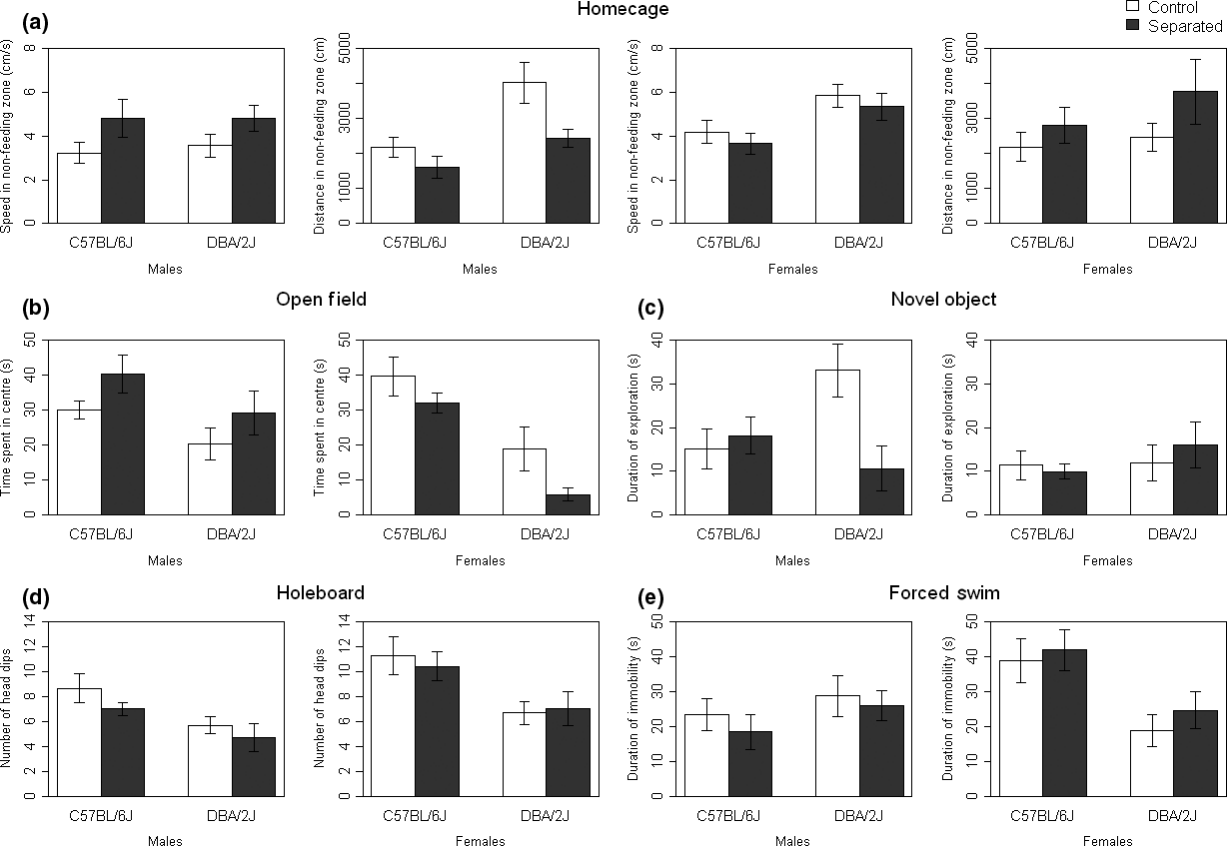
Behavioral tasks. Means (±SEM) for significantly different behavioral measures. (a) Maternally separated males differed from controls in speed (*P* < 0.05) and distance (*P* < 0.01) in the non-feeding zone of the homecage task. (b) Maternally separated males spend more time in the center of the open field (*P* < 0.05), whereas maternally separated DBA/2J females spend less time in the center (*P* < 0.05). (c) Increased exploration of the novel objects by maternally separated DBA/2J males (*P* < 0.05) but not females. (d) Measures from the holeboard task, with no significant differences between groups. (e) Measures from the forced swim task, with no significant differences between groups. Bars indicate maternally separated (black) or control (white) animals.

As expected, behavioral differences between sexes and strains were frequently observed in the different tests (see Fig. 1 for a detailed overview of the data), but not elaborated on here unless in the context of a difference between maternally separated and control animals.

#### Home cage

In the home cage test, significant differences between the maternally separated and control groups were only seen in male mice in the habituated dark hour (Fig. 1a), when mice are typically most active. Maternally separated males from both strains moved faster (E factor: *F*[1,34] = 5.4, *P* < 0.05) and over shorter distances (E factor: *F*[1,34] = 7.9, *P* < 0.01) in the non-feeding zone than controls.

#### Open field

Maternally separated mice reacted in a sex-specific way in the open field test (Fig. 1b). The time spent in the center of the arena was significantly greater in the MS male mice of both strains (E factor: *F*[1,40] = 4.3, *P* < 0.05) but significantly lower in the MS female mice from the DBA/2J strain only, demonstrating a genotype by environment interaction in an anxiety phenotype in response to MS (G × E interaction: *F*[1,36] = 5.1, *P* < 0.05).

#### Novel object exploration

Maternally separated DBA/2J male mice differed from controls in the novel object test, with no differences seen in C57BL/6J males, indicating another genotype by environment interaction in an exploratory phenotype in response to MS. The time spent exploring the novel object was significantly reduced in MS DBA/2J males compared to controls (G × E interaction: *F*[1,40] = 6.2, *P* < 0.05, Fig. 1c). There were no differences in exploration in the female mice of either strain.

#### Holeboard and forced swim

No significant differences were seen in either the holeboard or the forced swim tasks (Fig. 1d and e).

#### Corticosterone changes after MS

**Figure 2:**
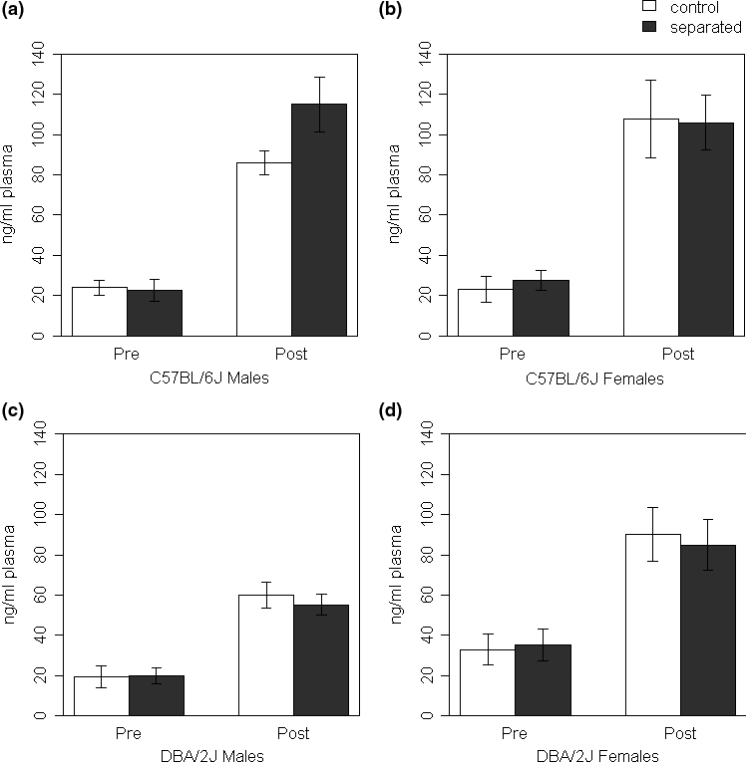
Corticosterone. Means (±SEM) for corticosterone levels pre- and post-forced swim stress. All groups display an increase in corticosterone levels post-stress (*P* < 2.2 × 10^16^). Maternally separated C57BL/6J males have a greater increase in corticosterone compared to controls following stress (*P* < 0.05). Bars indicate separated (black) or control (white) animals.

A significant increase above baseline in plasma corticosterone levels was seen in both strains and sexes in response to swim stress, regardless of experimental group (pre- to post-swim: *P* < 2.2 × 10^16^, Fig. 2). However, maternally separated C57BL/6J males had significantly higher corticosterone levels post forced swim compared to controls (G × E: *F*[1,40] = 4.3, *P* < 0.05, Fig. 2a), demonstrating a genotype by environment interaction in a physiological measure of the stress response (corticosterone) as a consequence of MS.

### DNA methylation changes in response to MS

#### *Avp*

**Figure 3:**
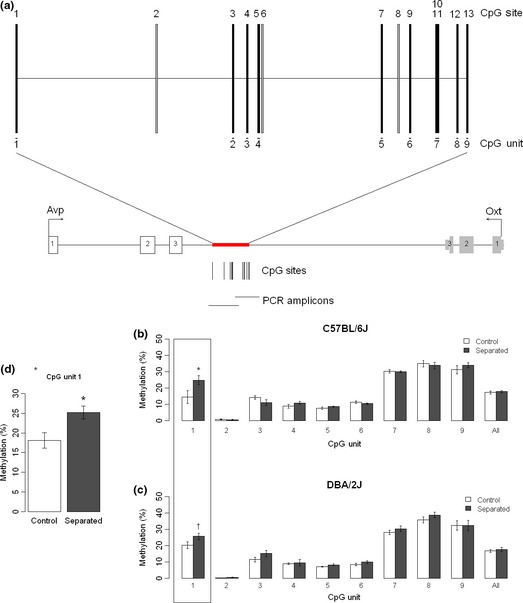
Avp. (a) Schematic diagram showing the Avp and Oxt (oxytocin) genes, orientated with the Avp gene reading forwards. Exons are indicated by the numbered boxes. The red box highlights the region assessed for DNA methylation in this study. Individual CpG sites are represented by black lines: gray lines show CpG sites unable to be assessed by our method. CpG units (individual sites or groups of sites) are indicated underneath the CpG sites. These unit numbers correspond to the graph showing mean (±SEM) methylation percentages. (b–c) Percentage methylation across CpG Units in the Avp CGI3 region. (d) Maternally separated males in both strains have increased methylation at CpG Unit 1 in Avp assay CGI3b (*P* < 0.05).

Two assays were designed to cover the region found to be differentially methylated in response to early life stress by Murgatroyd and co-workers (Murgatroyd et al. 2009), giving data for 9 CpG units spanning 10 CpG sites (Fig. 3a). Levels of methylation at specific CpG sites varied considerably across the region from 0.5 to 35.4%, with an amplicon average metC density of 17.2% (Fig. 3b and c). Maternally separated, male mice from both strains showed a significant increase in methylation at CpG Unit 1 (controls 18.1%, separated 25.2%, *P* < 0.05, Fig. 3d).

#### *Nr4a1*

**Figure 4:**
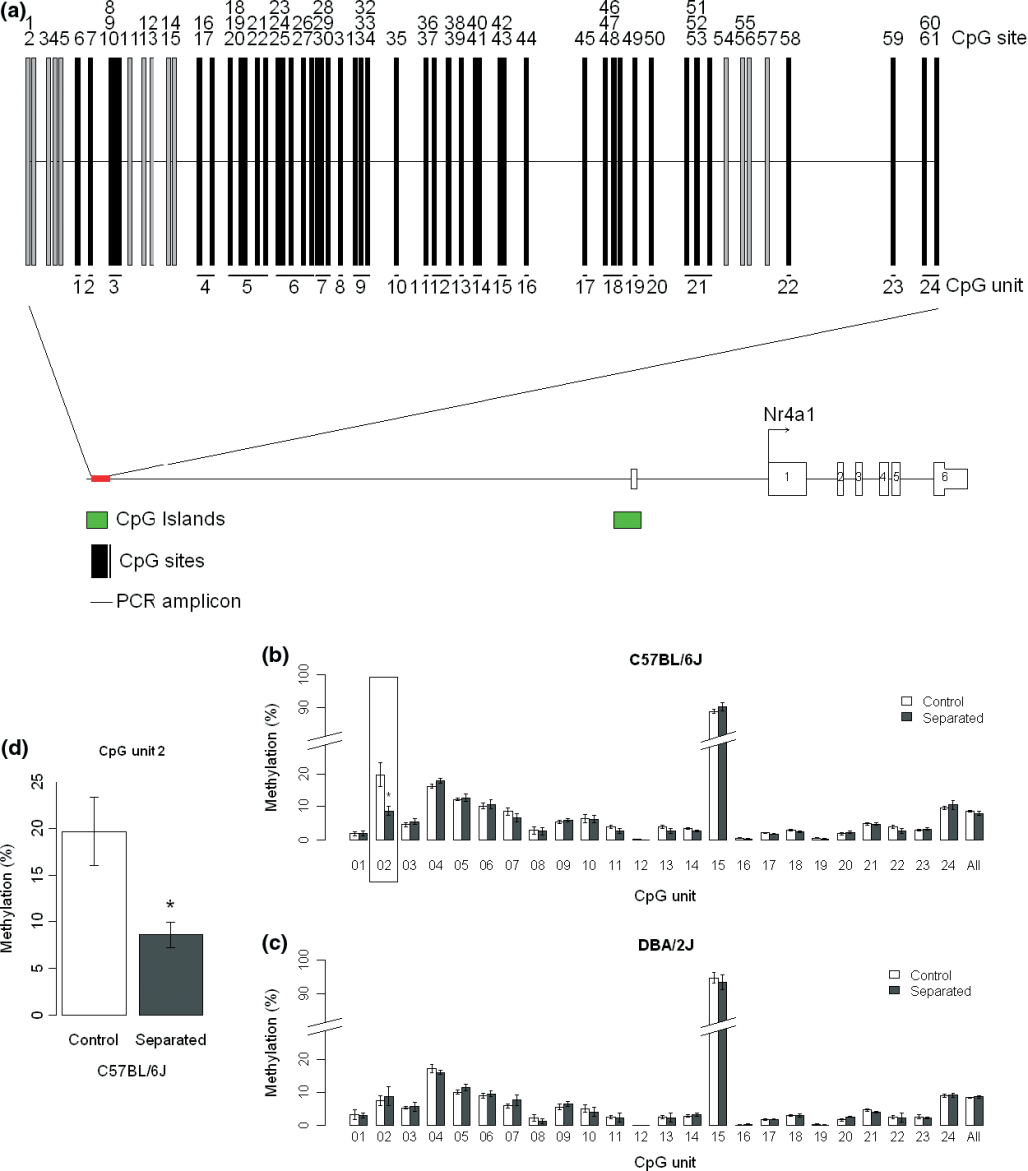
Nr4a1. (a) schematic diagram showing the Nr4a1 gene, orientated reading forwards. Exons are indicated by the numbered boxes. The red box highlights the region assessed for DNA methylation in this study. The green boxes indicate CpG islands in this region. Individual CpG sites are represented by black lines: gray lines show CpG sites unable to be assessed by our method. CpG units (individual sites or groups of sites) are indicated underneath the CpG sites. These unit numbers correspond to the graph showing mean (±SEM) methylation percentages. (b–c) Percentage methylation of a CpG region upstream of the Nr4a1 gene. (d) Maternally separated C57BL/6J males have decreased methylation at CpG Unit 2 (*P* < 0.01).

The assay gave reliable data for 24 CpG Units spanning 47 CpG sites (Fig. 4a). DNA methylation across the region was at a low level (amplicon average metC density = 4.9%) with the exception of CpG Unit 15, which had an average metC density of 91.9% (Fig. 4b and c). Maternally separated C57BL/6J males had decreased methylation at CpG Unit 2 (controls 19.7%, separated 8.8%, *P* < 0.01, Fig. 4d) but no differences were seen in the DBA/2J strain, demonstrating a genotype by environment interaction in DNA methylation levels following MS.

#### *Nr3c1*

**Figure 5:**
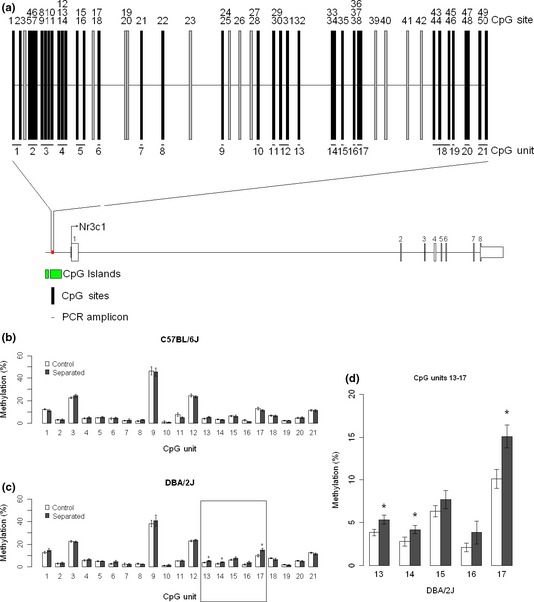
Nr3c1. (a) schematic diagram showing the Nr3c1 gene, orientated reading forwards. Exons are indicated by the numbered boxes. The red box highlights the region assessed for DNA methylation in this study. The green boxes indicate CpG islands in this region. Individual CpG sites are represented by black lines: gray lines show CpG sites unable to be assessed by our method. CpG units (individual sites or groups of sites) are indicated underneath the CpG sites. These unit numbers correspond to the graph showing mean (±SEM) methylation percentages. (b–c) Percentage methylation of a CpG region in the Nr3c1 gene. (d) Maternally separated DBA/2J males have increased DNA methylation at CpG Units 13 (*P* < 0.05), 14 (*P* < 0.05), and 17 (*P* < 0.01).

This assay gave reliable data for 21 CpG Units spanning 38 CpG sites (Fig. 5a). Overall, this region was characterized by low levels of DNA methylation (amplicon average metC density = 8.8%, Fig. 5b and c), with little between-individual variation. Maternally separated DBA/2J animals showed small but significant increases in DNA methylation at three CpG units within the Nr3c1 amplicon (Fig. 5d): CpG Unit 13 (controls 3.8%, separated 5.3%, *P* < 0.05), CpG Unit 14 (controls 2.8%, separated 4.2%, *P* < 0.05), and CpG Unit 17 (controls 10.1%, separated 15.1%, *P* < 0.01). No differences were seen in the C57BL/6J strain, indicating another genotype by environment effect on altered methylation.

## Discussion

This study investigated the phenotypic and DNA methylation changes in candidate genes resulting from early life exposure to stress. Our genetically informed design allowed us to determine whether phenotypic and/or epigenetic responses to adverse environmental exposures differed as a function of genetic background, as has previously been shown (Uchida et al. 2011). Analysis of corticosterone levels showed an altered physiological response to acute swim stress in maternally separated mice that was dependent on genetic background. In addition, early life stress induced a number of behavioral changes, many of which were sex- and strain-dependent, providing further support for an interaction between genetic background and exposure to an adverse environment. Finally, we observed altered DNA methylation in the hippocampus across promoter regions of three candidate genes, in maternally separated male mice compared to controls. Table 2 summarizes the results observed in this study.

**Table 2:** Summary of differences between maternally separated mice and controls

	Males		Females	
		
	C57BL/6J	DBA/2J	C57BL/6J	DBA/2J
Behavior				
Home cage activity (speed)	↑	↑	−	−
Home cage activity (distance)	↓	↓	−	−
Anxiety	↓	↓	−	↑
Exploratory activity	−	↓	−	−
Corticosterone level following stressor	↑	−	−	−
DNA methylation				
*Avp*	↑↑	↑		
*Nr4a1*	↓↓↓	−		
*Nr3c1*	−	↑		

No baseline differences in corticosterone were found between separated and control animals, and following the forced swim test all groups exhibited the expected significant increase in corticosterone (Fig. 2). Corticosterone is a stress hormone regularly measured in mice as a physiological indicator of stress (Barlow et al. 1975; Shanks et al. 1990) and following a stressful task, such as restraint or tail suspension tests, levels of the hormone have been shown to be significantly raised (Pitman et al. 1988). We observed a much greater increase in corticosterone levels following a stressful task in the C57BL/6J male separated mice, indicating that exposure to an early life stress had altered the physiological response to an acute stress which was strain-specific. This result reflects findings in the human literature that associate increased cortisol or corticotrophin-releasing hormone (CRH) with depressive illness (Gibbons and Mchugh 1962; Merali et al. 2004), and is consistent with the hypothesis that altered HPA activity can result from early life stress (Weinstock 1997).

Despite using the longer separation protocol, we found only mild changes in behavior following MS. Previous studies in both mice and humans have shown adverse behavioral phenotypes with a range of severity resulting from a number of early life stressors. In humans, early life stress has been shown to result in the development of depression, anxiety, schizophrenia, and post-traumatic stress disorder, along with a number of other psychiatric and physiological disorders (Agid et al. 1999; Morgan and Fisher 2007; Danese et al. 2009; Weich et al. 2009). Rodent models of early life stress have sought to establish a similar range of altered behaviors, and have been found to display a wide variety of phenotypes dependent on the stressor used, the severity of the stressful event and genetic background (Holmes et al. 2005). However, the behavioral effects generally tend to be mild unless a susceptible strain of mouse is used, such as BALB/cJ (Wang et al. 2011). The MS model employed in this study has been widely used in rats, and produces consistent behavioral alterations (e.g., Boccia and Pedersen 2001; Daniels et al. 2004; O'mahony et al. 2009). A shorter, repeated version of the MS model commonly used in mice can be less reliable, sometimes eliciting changes but with no consistent effects (Millstein and Holmes 2007), possibly due to the dams ability to adapt and provide compensatory care to the pups (Franklin et al. 2010). As we used the single 24 h MS model, we did not expect to elicit such compensatory behavior in the dams but as we did not assess maternal care, we cannot exclude a potential impact of altered maternal behavior in our study.

Our data find differences between MS and controls specifically in the home cage, open field and novel object exploration tests. As with previous findings (Eklund and Arborelius 2006; Renard et al. 2007), we found the effects of the MS model to be sex-dependent, with stronger effects observed in males. It is interesting to note that the strongest phenotypic differences occur mostly in males, while in human samples the prevalence of mental disorders such as depression is higher in females. MS male mice from both strains displayed altered activity characterized by rapid bursts of locomotor activity over short distances in the home cage and increased exploration of a novel arena. Increased exploration of a novel arena following early life stress has been reported previously in a number of studies (Daniels et al. 2009; Franklin et al. 2010) and has been suggested to be a result of hyperarousal or due to changes in stress reactivity. Conversely, DBA/2J female mice exposed to MS displayed reduced exploration of a novel arena, indicative of an anxiety-like behavior. Although DBA/2J males exposed to MS displayed increased exploration of a novel arena, they showed reduced exploration of novel objects, which suggests an altered response in threatening (novel arena) versus non-threatening (novel object) situations following MS.

We identified significant, but modest, changes in DNA methylation at CpG units in the promoter regions of three genes (*Avp*, *Nr4a1*, *Nr3c1*) following exposure to early life stress. In the *Avp* promoter, DNA methylation was increased at a single CpG Unit following MS in both strains of mice, with a >10% increase in the C57BL/6J strain. A decrease in DNA methylation following MS was seen at CpG Unit 2 in the *Nr4a1* gene only in C57BL/6J mice, whereas increases in methylation following MS at units 13, 14, and 17 in the *Nr3c1* gene were seen only in DBA/2J mice.

Previous research has demonstrated DNA methylation changes in a number of genes following early life stress. Epigenetic mechanisms may play a key role in the translation of environmental factors into phenotype. The epigenome is particularly susceptible to external disruption during a number of key developmental periods, and is thought most vulnerable to the effect of environmental factors during early development (Dolinoy et al. 2007). In this way, it is possible that epigenetic adaptations in response to early life adversity may play an important role in developmental plasticity, mediating phenotypic variability later in life (Bateson et al. 2004).

Our study found that a CpG site in an intergenic region near the *Avp* gene was hypermethylated in MS males in hippocampal tissue. A recent paper by Murgatroyd et al. (2009) found hypomethylation of multiple CpG sites in the same region, but only in tissue from the para-ventricular nucleus. These CpG sites, especially site 10, were associated with *Avp* expression differences, suggesting an important regulatory role for this region. We did not observe DNA methylation differences in any of the same sites as the Murgatroyd et al. (2009) study, although the discrepancies between the two studies may result from our use of a different brain tissue, and because the CpG sites interrogated in our assay did not overlap completely with those of Murgatroyd et al. (2009). For example, CpG Unit 2 in our assay, which corresponds to site 10 in the Murgatroyd study, was unable to be assessed and therefore we are unable to determine if there are DNA methylation differences between groups for that site.

However, some sites were assessed in both studies and this provides interesting directions to explore in future research. CpG Unit 1 in our study, which shows a significant hypermethylation in maternally separated animals, is shown to decrease in DNA methylation over time (from 6 week mice to 1 year mice) in the Murgatroyd et al. (2009) study, regardless of environment. This suggests that this site may be an area for variable DNA methylation, and in the case of the maternally separated animals a dysregulation of the decrease in DNA methylation over time could be a maladaptive response to early life stress.

We find modest hypermethylation across a number of sites in the promoter region of the glucocorticoid receptor following MS, specifically in DBA/2J mice. More marked hypermethylation in this region of the glucocorticoid receptor promoter following a stressful event have been reported by a number of groups (Weaver et al. 2004; Oberlander et al. 2008; Mcgowan et al. 2009), although these are not consistently found (Daniels et al. 2009). Weaver et al. (2004) observed that rat pups raised by mothers displaying reduced nursing behaviors (licking, grooming, and arched back nursing) demonstrate marked hypermethylation across a number of CpG sites, including a region containing an NGFI-a binding site (Weaver et al. 2004). We observed no differences in the site corresponding to the binding site (CpG unit 21), and saw much smaller differences in other significant sites from the Weaver study, suggesting that this region still warrants further investigation to clarify its role in early life stress.

Bioinformatic analysis has revealed a number of putative transcription factor binding sites within the regions examined in this study. In the *Nr4a1* assay, CpG site 2 is contained within a consensus sequence for cAMP response element-binding (CREB), and was found to be differentially methylated between MS and controls in C57BL/6J mice. CREB is a transcription element widely expressed in the brain (Berkowitz and Gilman 1990) and previously implicated in the regulation of brain-derived neurotrophic factor (BDNF, Tao et al. 1998) and corticotropin-releasing hormone (CRH, Yamamori et al. 2004), both of which have been shown to have expression changes following early life stress (Ladd et al. 1996; Roth et al. 2009). Previous studies have demonstrated that differential methylation status of CpG islands in CREB binding sites determine CREB binding and activity (Demura and Bulun 2008; Sunahori et al. 2009). This suggests a possible mechanism through which altered DNA methylation at this locus may influence transcription, although further study is needed to clarify whether this will effect gene expression via CREB.

Our study has a number of limitations which should be considered when interpreting the data. Firstly, it remains unknown whether DNA methylation changes of small magnitude at some CpG sites within a gene would have a functional effect, and the consequences of the DNA methylation differences on transcription levels will therefore need to be investigated in future studies. As gene expression levels are only indicative of the time point in which they were measured, and DNA methylation changes are thought to reflect a long-term reprogramming of the gene and gene expression, DNA methylation may not correlate if only measured at one time point. In addition, there is growing evidence for the plasticity of some DNA methylation sites over time, especially those involved in neuronal activation (Guo et al. 2011), but little is known about whether these would correlate with gene expression changes at a singular time point. The ideal study would therefore measure gene expression at a number of developmental stages, to determine the point at which DNA methylation differences have an effect. Secondly, to identify DNA methylation differences that may be relevant for the behavior changes seen a broader examination of the methylome is required than the results presented here. Finally, although we have determined an effect of early life stress on DNA methylation levels, this research would need to be repeated in females to uncover any sex effects. If the association of DNA methylation levels with behavior is sound, then we would expect to see much smaller DNA methylation differences at these sites in the females, in accordance with the behavior differences seen in females. We therefore cannot draw strong conclusions about the relationship between behavioral changes following early life stress and DNA methylation. Despite this, we provide compelling evidence that both behavior and DNA methylation in candidate genes differ following early life stress, and further research is needed to uncover the extent of causality between these two measures.

*Avp*, *Nr3c1*, and *Nr4a1* have all been shown to play a role in the regulation of the HPA axis. Our study finds increased DNA methylation of CpG sites in *Avp* and *Nr3c1*, and decreased methylation in Nr4a1. It is conceivable that differential methylation of these genes could result in dysregulation of the HPA axis during development, leading to altered stress behaviors in adulthood. In concordance with this, we find that MS mice showed differential stress reactivity in a number of behavioral tasks, and C57BL/6J mice experience a greater physiological stress response.

A key finding of our study is the effect of genetic background both on the behavioral and DNA methylation differences seen between groups. By using two different inbred strains of mice, we observed phenotypic and epigenetic changes that are potentially genotype-specific. It has previously been reported that inbred strains vary in their emotional and stress reactivity (Flint 2003; Lad et al. 2010), and additionally that their sensitivity to early life stress may vary to a similar extent (Holmes et al. 2005). Consistent with this, our results suggest that DBA/2J mice develop phenotypic changes to early life stress that are not seen in the C57BL/6J strain, whereas male C57BL/6J mice show an altered physiological response to stress following MS. Importantly, the DNA methylation differences found were also often strain-specific. Taken together, these findings highlight the importance of examining environmental effects on a range of genetic backgrounds, allowing the further dissection of environmental, genetic, and epigenetic interactions.
